# Surgical outcomes of extraskeletal myxoid chondrosarcoma

**DOI:** 10.55730/1300-0144.5422

**Published:** 2022-05-26

**Authors:** Coşkun ULUCAKÖY, İsmail Burak ATALAY, Aliekber YAPAR, Ahmet Yiğit KAPTAN, İzzet BİNGÖL, Mehmet DOĞAN, Mehmet Fatih EKŞİOĞLU

**Affiliations:** 1 Department of Orthopedics and Traumatology, Dr Abdurrahman Yurtaslan Ankara Oncology Training and Research Hospital, Ankara, Turkey; 2 Department of Orthopedics and Traumatology, Antalya Training and Research Hospital, Antalya, Turkey; 3 Department of Orthopedics and Traumatology, Harran University School of Medicine, Şanlıurfa, Turkey; 4 Department of Pathology, Dr Abdurrahman Yurtaslan Ankara Oncology Training and Research Hospital, Ankara, Turkey

**Keywords:** Extraskeletal, chondrosarcoma, recurrence, metastasis, tumor

## Abstract

**Background/aim:**

Extraskeletal myxoid chondrosarcoma (EMC) is a rare soft tissue sarcoma. The aim of this study is to present the results of the patients we treated with the diagnosis of EMC as an oncology reference center.

**Materials and methods:**

Information on 13 patients diagnosed with EMC between 2006 and 2018 was retrospectively reviewed. Patients’ demographic information, tumor sizes, surgical treatments, chemotherapy and radiotherapy statuses, follow-up times, recurrences, and metastases were recorded.

**Results:**

Mean patient age was 53.6 ± 15 years (range: 28–73). In 8 patients, the tumor was located in the lower limbs, most commonly in the thigh (46.2%). Mean follow-up period was 52.8 ± 19.9 (24–96) months. All patients underwent wide resections and only one had a positive surgical margin. In follow-up, 5 (38.5%) patients experienced recurrence; 6 patients had lung metastasis (46.2%) and 7 patients (53.8%) died. Mean tumor size was 10.4 ± 3.2 (5–17) cm. Median survival time was 61 (50.5–71.4) months and 5-year survival rate was 51.8%. There was no significant difference between survival times according to age, gender, side, limb location, postoperative radiotherapy, recurrence, or presence of lung metastasis. The cut-off value for death obtained by ROC analysis of tumor size was 11 cm.

**Conclusion:**

EMC is a rare soft tissue sarcoma with high local recurrence and metastasis capacity. Tumor size and metastatic disease are poor prognostic criteria. If it is a localized disease, the first option should be wide resection.

## 1. Introduction

Extraskeletal myxoid chondrosarcoma (EMC) is a rare soft tissue sarcoma that accounts for 2.5% to 3% of all soft tissue sarcomas. EMC is characterized by a multinodular architecture, myxoid matrix, and malignant chondroblasts [1,2]. Enzinger and Shiraki were the first to describe it in 1972, and it was accepted as a low-grade lesion in the first years due to its histological appearance [3]. In the following years, Oliveira et al. [4] defined EMC as a separate sarcoma of chondroblastic origin originating from extraskeletal soft tissues. However, there is no consensus in the literature regarding the histopathogenesis of EMC, and EMC is currently classified as a vague differentiation tumor in the revised version of the World Health Organization (WHO) Classification of Tumours of Soft Tissue and Bone [5]. Furthermore, a definitive distinction from other myxoid tumors is difficult because EMC has no pathognomonic clinical, imaging, or pathological characteristics. EMC is often found in the proximal extremities of middle-aged men and is usually asymptomatic; it has a slow course but a high tendency for local recurrence and metastasis [6]. Although their course is slow, these tumors have a significant risk of eventual relapse and metastasis, especially for the lungs. The current approach in the treatment of EMC is primarily surgical treatment if the disease is localized, and radiotherapy if necessary. If the disease is advanced rather than localized, antiangiogenic agents, besides standard chemotherapy, have recently shown promising activity. Although the expected recurrence risk is approximately 50%, it is the prolongation of the desired life expectancy [7]. Because of the very low incidence, there are no clinical studies investigating the best treatment options for these tumors. The aim of this study is to present the results of the patients we treated with the diagnosis of EMC as an oncology reference center.

## 2. Materials and methods

**Figure 1:**
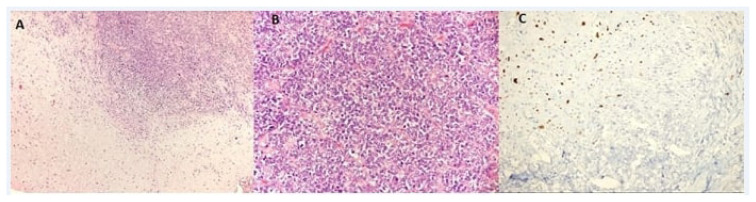
A) Combined view of undifferentiated areas of hyaline cartilage and small cells in the tumor (magnification 100×, H&E); B) Small undifferentiated cell component (magnification 400×, H&E); C) Immunohistochemical S100 positivity in cartilaginous areas (magnification 200×).

The data of 13 patients diagnosed with EMC in our clinic between January 2006 and January 2018 were retrospectively reviewed. Demographic information, tumor sizes, surgical treatments, chemotherapy and radiotherapy statuses, follow-up periods, recurrences, and metastases of the patients were recorded. Histopathologically, EMC is characterized by abundant hypocellular myxoid matrix and interconnected cords of uniform neoplastic cells with a common spindle cell differentiation. Patients whose diagnosis of EMC was confirmed histopathologically were included in the study (different immunohistochemical images of EMC Figures 1A–1C). For the patients whose diagnosis of EMC was confirmed, a multidisciplinary decision was made in the tumor council of our hospital in accordance with the current treatment principle, and the treatment option (surgical treatment, chemotherapy, and radiotherapy) was decided. Patients whose diagnosis could not be established pathologically and whose file information could not be obtained were excluded from the study. The study protocol was approved by the institutional review board of Dr Abdurrahman Yurtaslan Oncology Hospital (2020/103).

### 2.1. Statistical analyses

Statistical analyses were performed using IBM SPSS Statistics for Windows, version 22.0 (IBM Corp., Armonk, NY, USA). Descriptive statistics were presented as numbers and percentages for categorical variables and as mean ± standard deviation and median (minimum–maximum values) for continuous variables. Prognostic values of tumor size were assessed using receiver operating curve (ROC) analysis. The area under the ROC curve (AUC) results were considered excellent for AUC values of 0.9–1, good for AUC values of 0.8–0.9, fair for AUC values of 0.7–0.8, poor for AUC values of 0.6–0.7, and failed for AUC values of 0.5–0.6. Following ROC analysis, AUC and cut-off values, sensitivity and specificity of those cut-off values, likelihood ratios, and positive predictive values and negative predictive values were presented. Survival analyses were performed with the Kaplan–Meier method and log-rank test. Values of p < 0.05 were considered statistically significant [8,9].

## 3. Results

Thirteen patients diagnosed with EMC with a mean age of 53.6 ± 15 (range: 28 to 73) years were included in this study (Table 1). In 8 of the patients, the tumor was located in the lower limbs and the most common location in the lower extremities was the thigh (46.2%). The mean follow-up period of the patients was 52.8 ± 19.9 (range: 24 to 96) months. Wide resection was performed in all patients and only 1 patient had a positive surgical margin. While recurrence developed in 5 (38.5%) of the patients during follow-up, lung metastasis (46.2%) was detected in 6 patients, and 7 patients (53.8%) died due to the disease and complications related to the disease. The average tumor size was determined as 10.4 ± 3.2 (range: 5 to 17) cm (Table 2).

**Figure 2:**
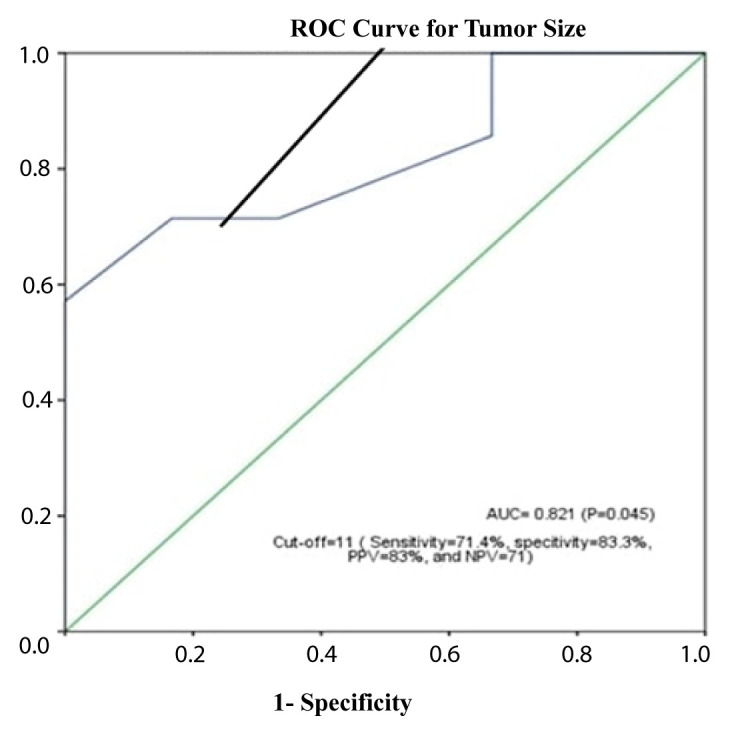
Receiver operating characteristic (ROC) curves for tumor size (PPV: positive predictive value, NPV: negative predictive value).

**Figure 3:**
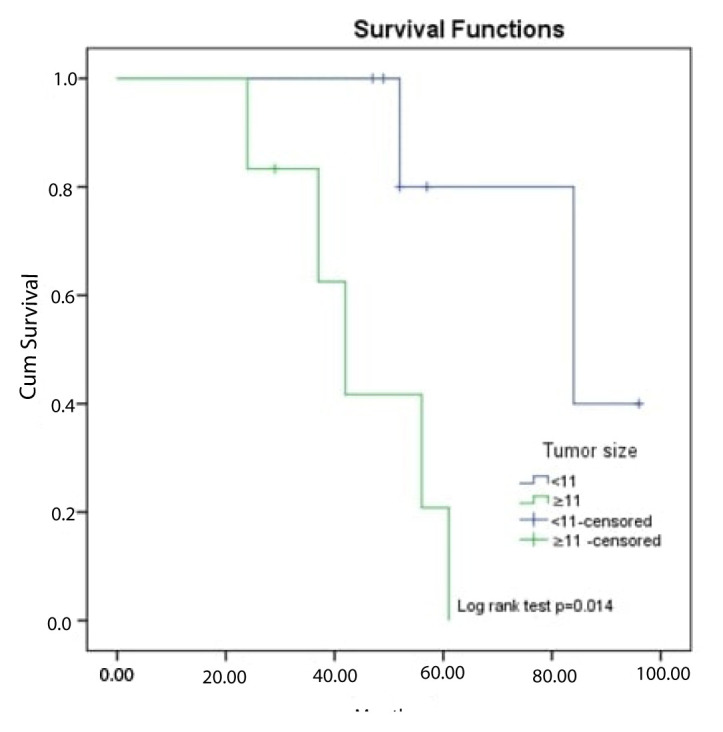
OS curve of patients according to tumor size groups.

**Figure 4:**
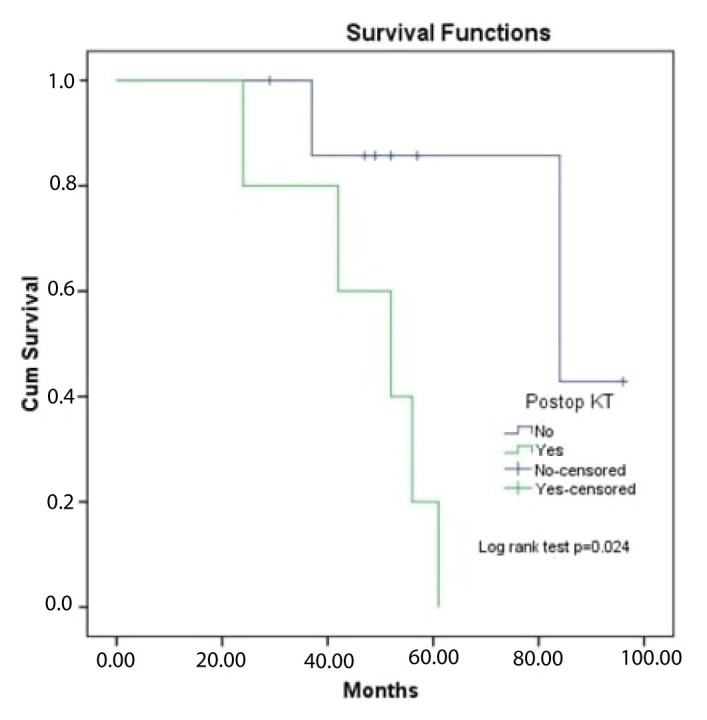
OS curve of patients according to CT groups.

The median survival time of the patients in the study was 61 (range: 50.5–71.4) months. The 5-year survival rate was 51.8%. It was found that there was no significant difference between survival times according to age, sex, side, extremity location, receiving postoperative radiotherapy, recurrence, and presence of lung metastasis (log-rank tests: p > 0.05) (Table 3). The cut-off value for death obtained by ROC analysis of the tumor size was 11 cm (Figure 2). Accordingly, it was observed that the survival times of patients with a tumor size of ≥11 were statistically significantly shorter (log-rank test: p = 0.014) (Figure 3). It was also determined that the survival times of patients who received postoperative chemotherapy (metastatic patients) were significantly shorter than those of patients who did not (log-rank test: p = 0.024) (Figure 4).

## 4. Discussion

EMC is a rare soft tissue tumor that occurs twice as often in males than in females in the extremities of middle-aged adults [10]. The staging system of the French Federation of Cancer Centers (FNCLCC) lists EMC as a stage 2/3 tumor [11]. Our knowledge about tumor molecular features has increased in recent years; it is now known that it harbors the tumor translocation (9; 22) (q22; q11) and results in the EWSR1/NR4A3 sequence in most patients [5,12]. Other translocations have also been identified. The main finding of this study is that EMC is a rare malignant soft tissue sarcoma with high recurrence and lung metastasis capacity. In addition, metastasis and tumor size are poor prognostic factors.

Since EMC has no distinctive clinical features, its differential diagnosis is difficult and it may be confused with other myxoid soft tissue sarcomas, such as myxofibrosarcoma, myxoid liposarcoma, and metastatic carcinomas, including myoepithelial tumors and neuroendocrine tumors. Usually the tumor presents as a single slow-growing soft tissue lesion, about 5–10 cm in diameter [1]. With computed tomography, EMCs appear as soft tissue masses with lobular contours [1]. Oliveria et al. [4] presented the results of 23 EMC cases at the Mayo Clinic in 2000; the mean tumor size of the patients with a mean age of 50 was 9.5 cm and tumors were located in the lower extremities in 83% of the patients. In addition, Oliveria et al. [4] showed that tumor sizes greater than 10 cm had worse prognosis. Similarly, Mcgrory et al. [13] said that tumor size is a poor prognostic factor. In our study, conducted with 13 EMC patients with a mean age of 53.6, tumors were most frequently located in the lower extremities (61.5%) and the mean tumor size was 10.4 cm. Tumor sizes above 11 cm were found to be associated with mortality.

Since EMCs are rare tumors, our knowledge about this disease is limited, making it difficult to draw definitive conclusions about the clinical features, prognostic factors, and appropriate treatment of the tumors. Initially, it was considered by Enzinger and Shiraki as a low-grade sarcoma according to the initial definition of the tumor, and only 4 out of 34 patients were reported to have died of the disease in short-term follow-up (median: 3.5 years) [3]. Later, Saleh et al. [14] showed that EMC was a destructive tumor with high local recurrence and metastasis capacity among the 10 EMC patients they followed. Similarly, Ogura et al. [15] found 31% recurrence and 50% lung metastasis in the 22 EMC patients they followed. Likewise, we found high rates of local recurrence (38.5%) and metastasis (46.2%) in 13 patients followed by EMC.

Despite its high recurrence and metastasis rates, there are publications in the literature showing that EMC is a slow-progressing sarcoma with 5-year survival rates between 70% and 100% [4, 12, 13]. Our 5-year survival rate (51.8%) contradicts the findings in the literature. We attribute this to our encounter with an advanced patient group rather than a localized disease, since we are an oncology reference center in our country.

EMC has limited response to chemotherapy; neither single nor combined chemotherapeutic agents showed statistically significant results in treating EMC and more research for identifying novel target therapies is therefore necessary [12]. In addition, the current approach does not recommend chemotherapy in localized disease. Therefore, the group receiving chemotherapy is the group with advanced disease with a lower life expectancy [7]. Similarly, in our study, there was no positive contribution to the life expectancy of patients receiving postoperative chemotherapy.

This study has some limitations. Firstly, it is a single-center retrospective study. In addition, the number of patients was small because EMC is a rare tumor. Not checking scores for functional outcomes was another limitation. All the same, this study is valuable due to the limited number of publications on the surgical and clinical results of EMC.

## 5. Conclusion

EMC is a rare soft tissue sarcoma with high local recurrence and metastasis capacity. Tumor size and metastatic disease are poor prognostic criteria. If it is a localized disease, the first option should be wide resection.

## Figures and Tables

**Table 1:** Information about patients.

No	Age	Localization	Tumor size, cm	Surgical border	RT CT	Recurrence	Lung metastasis	Follow-up time (n)	Exitus
1	53	Elbow	5	−	RT	−	−	86	
2	74	Elbow	12	+	RT	+	−	27	Ex
3	28	Shoulder	11	−	RT	−	−	19	
4	62	Shoulder	8	−	RT, CT	+	+	42	Ex
5	66	Wrist	11	−	CT	−	+	14	Ex
6	44	Cruris	14	−	CT	+	+	32	Ex
7	43	Cruris	9	−	−	+	−	37	
8	52	Thigh	17	−	CT	−	+	46	Ex
9	58	Thigh	7	−	RT	−	−	42	
10	67	Thigh	9	−	RT	+	−	47	
11	34	Thigh	13	−	CT	−	+	51	Ex
12	41	Thigh	10	−	−	−	−	39	
13	75	Thigh	9	−	−	−	+	74	Ex

RT: radiotherapy; CT: chemotherapy n: month

**Table 2:** Baseline data of malignancies.

Characteristics	Total N = 13
Sex, n (%)	
Female	5 (38.5)
Male	8 (61.5)
Age, years	
Mean ± SD	53.6 ± 15
Median(min–max)	53 (28–75)
Side, n (%)	
Right	6 (46.2)
Left	7 (53.8)
Extremity, n (%)	
Upper	5 (38.5)
Lower	8 (61.5)
Localization, n (%)	
Thigh	6 (46.2)
Cruris	2 (15.4)
Shoulder	2 (15.4)
Elbow	2 (15.4)
Wrist	1 (7.7)
Follow-up time, months	
Mean ± SD	52.8 ± 19.9
Median(min–max)	52 (24–96)
Tumor size, cm	
Mean ± SD	10.4 ± 3.2
Median (min–max)	10 (5–17)
Exitus, n (%)	
Yes	7 (53.8)
No	6 (46.2)
Surgical treatment, n (%)	
Wide resection	13 (100)
Surgical margin, n (%)	
Positive	1 (7.7)
Negative	12 (92.3)
Postoperative radiotherapy	
Yes	6 (46.2)
No	7 (53.8)
Postoperative chemotherapy	
Yes	5 (38.5)
No	8 (61.5)
Recurrence, n (%)	
Yes*	5 (38.5)
No	8 (61.5)
Lung metastasis	
Yes	6 (46.2)
No	7 (53.8)

* Recurrence surgery: 2 patients underwent wide resection, 3 patients underwent amputation

**Table 3:** Overall survival rates according to selected variables.

Total N = 13	Log-rank test p	Overall survival, months, Median (95% CI)	3-year survival rate, %	5-year survival rate, %
All patients		61 (50.5–71.4)	92.3	51.8
Sex	0.911			
Female		61 (32.6–89.4)	100	75
Male		56 (47.5–64.5)	87.5	56.3
Age	0.635			
<53 (median age)		56 (35.7–76.4)	100	40
≥53		84 (35.1–132.9)	85.7	57.1
Side	0.653			
Right		52 (28.9–75.1)	83.3	31.3
Left		61 (53.6–68.6)	100	64.3
Extremity	0.491			
Upper		52 (28.8–75.2)	80.0	26.7
Lower		61 (53.4–68.6)	100	65.6
Tumor size				
<11 cm		84 (37.4–130.5)	100	80
≥11 cm	0.014	42 (31.6–52.4)	83.3	20.8
Radiotherapy	0.389			
Yes		75 (52.9–97.9)	100	60
No		56 (38.4–73.6)	85.7	47.6
Chemotherapy	0.024			
Yes		52 (30.5–73.4)	80	20
No		84 (17.3–150.7)	100	85.7
Recurrence	0.222			
Yes		52 (36.4–67.6)	100	30
No		61 (50.8–71.1)	87.5	43.8
Lung metastasis	0.085			
Yes		52 (35.2–68.8)	83.3	33.3
No		86 (68.5–103.7)	100	83.3
